# Internally Formed Preferences for Options only Influence Initial Decisions in Gambling Tasks, while the Gambling Outcomes do not Alter these Preferences

**DOI:** 10.1007/s10899-024-10326-2

**Published:** 2024-06-26

**Authors:** Jianhong Zhu, Kentaro Katahira, Makoto Hirakawa, Takashi Nakao

**Affiliations:** 1 Graduate School of Humanities and Social Sciences, Hiroshima University, 1-1-1 Kagamiyama, Higashi-hiroshima, Hiroshima, 739-8524 Japan; 2 Department of Cognitive and Psychological Sciences, Graduate School of Informatics, Nagoya University, Furo, Chikusa, Nagoya, Aichi 464-8601 Japan

**Keywords:** Preference, Gambling, Internally guided decision-making, Externally guided decision-making, Computational modeling

## Abstract

All humans must engage in decision-making. Decision-making processes can be broadly classified into internally guided decision-making (IDM), which is determined by individuals’ internal value criteria, such as preference, or externally guided decision-making (EDM), which is determined by environmental external value criteria, such as monetary rewards. However, real-life decisions are never made simply using one kind of decision-making, and the relationship between IDM and EDM remains unclear. This study had individuals perform gambling tasks requiring the EDM using stimuli that formed preferences through the preference judgment task as the IDM. Computational model analysis revealed that strong preferences in the IDM affected initial choice behavior in the EDM. Moreover, through the analysis of the subjective preference evaluation after the gambling tasks, we found that even when stimuli that were preferred in the IDM were perceived as less valuable in the EDM, the preference for IDM was maintained after EDM. These results indicate that although internal criteria, such as preferences, influence EDM, the results show that internal and external criteria differ.

**Supplementary Information:**

The online version contains supplementary material available at 10.1007/s10899-024-10326-2.

## Introduction

When holding complimentary casino chips, some people choose to utilize the chips to select games with higher winning probabilities to obtain monetary rewards, while others opt to use the chips to experience the games they desire to play. The former behavior is akin to gambling, characterized by choosing the correct answer from the external environment under uncertain conditions, namely choosing games that would yield high returns (i.e., win) and adjusting decisions based on past experiences to obtain rewards provided by the external environment, known as externally guided decision-making (EDM). The latter is a choice based on internal values (such as preferences and beliefs) rather than answers provided by the external environment, termed internally guided decision-making (IDM) (Nakao et al., 2012, 2013, 2016, 2019; Ugazio et al., 2021; Wolff et al., 2019). These two decision-making types differ in conceptual and operational definitions and neural bases (Nakao et al., 2012, 2013, 2016, 2019; Ugazio et al., 2021; Wolff et al., 2019) and are considered to employ different decision-making processes.

Similar reward-related neural responses have been reported in studies on both EDM (Bechara et al., 1997, 2005; Gläscher et al., 2012; Marco-Pallarés et al., 2008, 2015; Mas-Herrero et al., 2015; Yacubian et al., 2006) and IDM (Aridan et al., 2019; Camille et al., 2011; Fellows & Farah, 2007; Izuma et al., 2010; Miyagi et al., 2017; Nakao et al., 2016). Moreover, a previous study showed that the values learned through the EDM in gambling tasks affected those formed through the IDM in preference choices (Zhu et al., 2024). It is possible that those who combine EDM and IDM do not base their decisions on entirely different criteria and that there are commonalities in the criteria referenced during these two types of decision-making.

Although Zhu et al. (2024) demonstrated the value learned through EDM affects IDM, it is unknown whether the values learned in IDM impact EDM. Similar to the gaming experience mentioned earlier in the casino, even in a gambling scenario, individuals may still rely on their preferences to make judgments, indicating a potential interplay between the EDM and IDM in various contexts. Additionally, even if the values learned in IDM affect EDM, this does not necessarily reflect that the internal and external criteria are represented as the same criteria and may mean that the different criteria, internal and external criteria, influence each other.

To shed more light on the relationship between the learning of values in gambling tasks and the preferences formed through free choice, this study aims to clarify whether preferences formed in the IDM influence behavior in gambling tasks that require the EDM. Furthermore, by examining whether the value learned in the EDM alters preferences already formed by the IDM, we determine whether internal and external criteria are represented differently.

To that end, we implemented the experiments in the order of the preference judgment task as the IDM, the gambling task requiring the EDM, and the subjective preference evaluation task. We used novel contour shapes, which can be regarded as having the same initial preference (Kunisato et al., 2012; Ohira et al., 2009, 2010), in these tasks to avoid confounding the effects of various types of emotions induced by items with daily formed preferences (e.g., Loewenstein & Lerner, 2003). By performing the preference judgment task on novel contour shapes, we made participants learn IDM values (i.e., preferences) within the experiment and examined whether these values affect EDM or are maintained after EDM (see the next paragraph for values learned in the IDM). We did not measure subjective preference before the preference judgment task because people cannot be certain of their subjective preferences for items they have never seen (Berlyne, 1970). Therefore, we needed to clarify participants’ preferences through repeated choices in the IDM. Preference for each stimulus was assessed from the chosen frequency in the IDM (preference judgment) by following previous studies (Di Domenico et al., 2013, 2015, 2016; Nakao et al., 2010, 2013, 2019; Zhu et al., 2021, 2024).

It is well known that choosing a behavior without accompanying feedback changes preferences for the chosen path and causes items in preference judgments to be rejected, which is called “choice-induced preference change” in IDM (Brehm, 1956; Colosio et al., 2017; Miyagi et al., 2017). More specifically, the preference for the chosen and rejected items increases and decreases, respectively. Zhu et al. (2021) reported that choice-induced preference change is observed in a preference judgment task (i.e., forced choices of choosing the preferred one from two paired items) without subjective evaluation. Thus, we used the same preference judgment task as Zhu et al. (2021) to estimate participants’ preferences for novel contour shapes.

For the gambling task, in addition to novel stimulus pairs that were not presented in the IDM, we used the most frequently and least frequently chosen stimulus pairs. There were two types of gambling tasks: one where the preferred stimulus had a high probability of receiving a reward and the other where the preferred stimulus had a low probability of receiving a reward. In these tasks, the preferred stimulus identified in the IDM was matched with stimuli of high and low probability in the EDM, respectively. To determine whether preferences in IDM affect EDM, we compared the correct response rates for novel and IDM stimulus pairs for the gambling tasks. By applying computational model analysis to the data of gambling tasks, we investigated whether preferences in IDM were reflected in the initial value of EDM and whether the reflected value was sustained in the subsequent EDM trials. Furthermore, by comparing the subjective preferences of novel stimuli and stimuli with IDM preference in the gambling tasks, we examined whether preferences learned in IDM are still retained after value learning in EDM.

For estimating the value in EDM, we used the reinforcement learning (RL) model (Sutton & Barto, 1998; Watkins & Dayan, 1992), which has been widely used to explain the value-learning mechanism in EDM (Daw & Doya, 2006; Dayan & Abbott, 2005; Dayan & Balleine, 2002; Gläscher et al., 2010; Schönberg et al., 2007). The value of the chosen option is updated based on the difference between the expected value of item and the actual feedback (Behrens et al., 2007; Biele et al., 2011; Gluth et al., 2014; Hauser et al., 2015; Katahira et al., 2011; Lindström et al., 2014; O’Doherty et al., 2007). Based on the basic RL model, we constructed four RL models with different assumptions about the types of stimuli that reflect preferences in IDM and applied them to behavioral data in EDM. We compared models to detect which model fit best with the behavioral data and used that model to estimate stimulus values.

## Method

### Participants

The behavioral experiment involved 42 healthy Japanese university students (male = 14, female = 28, mean age = 20.64, *SD* = 1.86), all of whom were native Japanese speakers.

### Stimuli and Apparatus

We selected 18 novel contour shapes from the Endo et al. (2003) study. The physical characteristics of these shapes were similar, with moderate values for smoothness (*M* = 4.48, *SD* = 1.66), complexity (*M* = 5.19, *SD* = 1.45), orientation (*M* = 4.96, *SD* = 2.06), symmetry (*M* = 3.72, *SD* = 1.77), width (*M* = 5.65, *SD* = 1.79), and association (*M* = 64.40, *SD* = 7.03). These shapes appeared in PsychoPy (Peirce et al., 2019) on a Windows 10 computer with a 1,920 × 1,080 pixels monitor.

### Design and Procedure

**Fig. 1:**
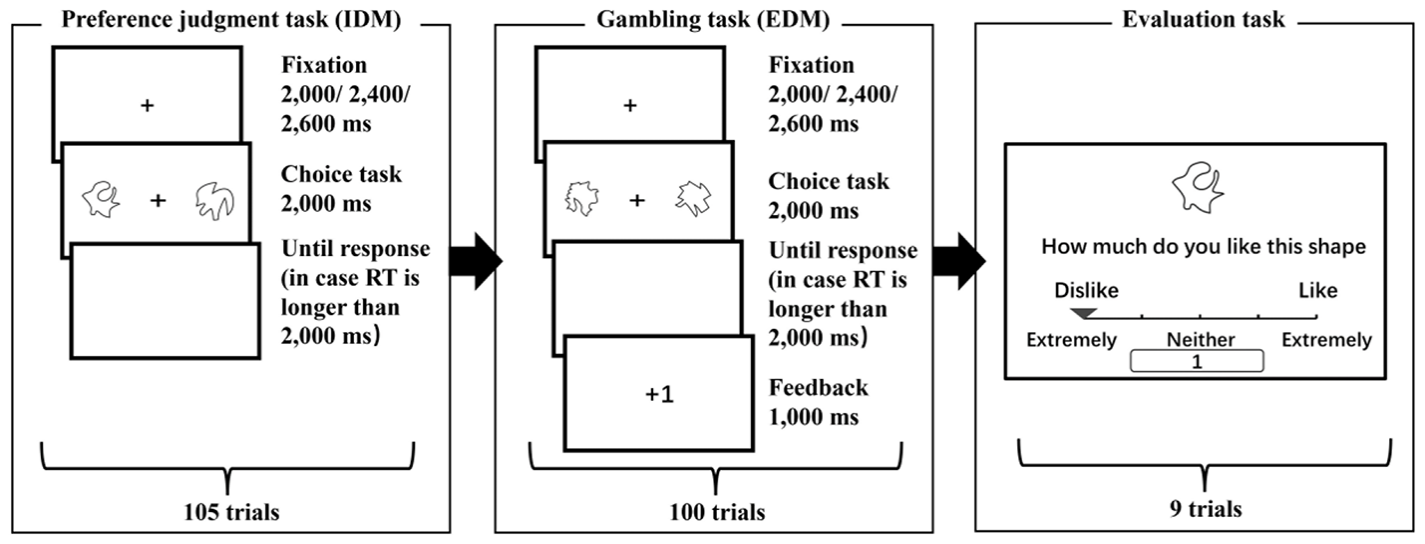
Experimental flow of each behavioral task. Behavior tasks were performed twice in the order of a preference judgment task, a gambling task, and a subjective preference evaluation task. In each preference judgment task and gambling task, participants were asked to select their preferred shape and the correct shape from a set of two shapes, respectively. There was no feedback presented for the preference judgment task. Participants’ subjective preference evaluations for all the shapes used in one behavioral task were collected after each gambling task

The IDM (preference judgment) task, EDM (gambling) task, and subjective preference evaluation were used as one task set (Fig. 1) and performed twice. The two task sets differed in terms of whether the most preferred stimulus in the IDM was assigned as a high probability or a low probability reward stimulus in the EDM. The stimuli used in the two task sets also differed.

In each preference judgment task, 105 stimulus combinations for 105 trials were created from seven different stimuli and divided into blocks consisting of 21 trials. Each stimulus was presented 30 times, and the stimulus combinations differed within each block. We asked participants to choose a preferred shape in each trial according to their own criteria.

The EDM included the high (the most selected) and low (the least selected) preference stimulus pairs in each preference judgment task, as well as one novel stimulus pair. Each stimulus pair was randomly presented 50 times. Participants were instructed to select one of the two correct shapes as quickly and accurately as possible. Correct and incorrect answers were rewarded 1 and 0 points, respectively. The probability of correct shapes was set at 70% vs. 30% for all pairs. Besides, the probability of reward for the two stimuli in each stimulus pair was independent of each other. Participants were informed that the more points they earned, the higher the honorarium they would receive after the experiment. When selecting any stimulus in each stimulus pair, there was a probability of earning 1 point regardless of the answer selected; however, we did not tell them the specific probability. The probability of rewarding a stimulus pair used in the preference judgment task was opposite in the two gambling tasks: whereas high preference stimulus has a 70% probability of being rewarded in a gambling task, which we call a “preferred stimulus high probability task”, IDM’s preferred stimulus has a 30% probability, which we call a “preferred stimulus low probability task.” The execution order of these two types of tasks was balanced among the participants.

For both the preference judgment and gambling tasks, each trial started with a fixation cross displayed for 2,000 ms, 2,400 ms, or 2,600 ms at random, followed by one stimulus pair displayed for 2,000 ms. The left and right stimuli in a trial were presented in random order. Participants used the standard keyboard’s F and J keys to select the stimuli on the left and right, respectively. If they did not complete the response within the 2,000 ms stimulus presentation, a white screen was displayed until the participant responded to control the shapes’ exposure period. In the gambling tasks, feedback was displayed for 1,000 ms after the response was made.

Finally, as the subjective preference evaluation task, participants were asked to rate their subjective preference for each shape on a 5-point Likert scale (1 = Extremely disliked, 5 = Extremely liked).

### Behavioral Data Analysis without Computational Models

To ensure that preferences differ between stimuli with high and low preferences, after the preference judgment task, we averaged the chosen frequencies of each person in the two preference judgment tasks and compared the chosen frequencies of high and low preference stimuli through a *t*-test.

To investigate whether the value (preference) of learning in IDM has an effect on EDM, we used analysis of variance (ANOVA) to compare the correct response rates of each stimulus pair in the gambling tasks.

Moreover, to compare the subjective preferences of the four stimuli in each gambling task, we used the multiple comparison procedure (Holm’s method) with *t*-tests.

### Computational Models

To determine the effect of preferences learned in the IDM on the EDM, we prepared four RL models (see Table 1) with different initial stimuli values. These stimuli include high and low preference formed in IDM and novel stimuli in EDM, respectively. RL 1 indicated no influence of the preference formed in the IDM, and the initial value was estimated by a free parameter *η* (0 ≦ *η* ≦ 1), which was the same for all stimuli in the EDM. RL 2 denoted that only the initial value of the high preference stimulus could reflect the preference in IDM and differed from the other stimuli in EDM. The initial value of high preference stimulus was estimated by *η1*, whereas the others were estimated by *η2*. RL 3 contrasted with RL 2, in which only low preference differed from the others. RL 4 represented the initial value of both the high and low preference stimuli that could reflect preferences in IDM. In the RL 4, all stimuli had different initial values and were estimated with different free parameters.

**Table 1:** Parameter settings of initial values of each stimulus in the EDM in different RL models

Model	Novel	High preference	Low preference
RL 1	*η*	*η*	*η*
RL 2	*η*_*2*_	*η*_*1*_	*η*_*2*_
RL 3	*η*_*1*_	*η*_*1*_	*η*_*2*_
RL 4	*η*_*3*_	*η*_*1*_	*η*_*2*_

*Note* “High preference” and “Low preference” represent the stimuli with high and low preferences in the IDM, respectively. “Novel” indicates novel stimuli in the EDM that were not used in the IDM. *η* is the free parameter for estimating the initial value of each stimulus

All models were based on the typical RL model (Watkins & Dayan, 1992; Eq. 1). In each trial *t*, the RL model updated the value *Q* (0 ≦ *Q* ≦ 1) of the chosen option based on the learning rate *α* (0 ≦ *α* ≦ 1) and feedback *r* (0 or 1). The learning rate determines the degree of value change, whereas the value of the rejected option remains unchanged until it is chosen again.

1 $${Q}_{i}\left(t+1\right)=\left\{\begin{array}{c}{Q}_{i}\left(t\right)+\alpha \left(r\left(\text{t}\right)-{Q}_{i}\left(t\right)\right) if i was chosen\\ { Q}_{i}\left(t\right) if i was rejected\end{array}\right.$$

Given the value of two options, the probability of choosing one option in a trial is calculated using the Softmax function (Eq. 2), where the slope of the Softmax function is determined by inverse temperature *β* (0 ≦ *β* ≦ 1).

2 $${P}_{chosen}=\frac{1}{1+\text{e}\text{x}\text{p}(-\beta ({Q}_{chosen}\left(t\right)-{Q}_{rejected}\left(t\right))}$$

### Computational Model Analysis of Actual Experimental Data

We applied all RL models to the integrated data from the gambling tasks to conduct a computational model analysis of the actual behavioral data in the gambling tasks. The results of this comparison were used to determine whether the preferences in IDM would be reflected in the initial stages of EDM. We then estimated and compared the value of each stimulus in EDM using the model that best fits the behavioral data. To confirm that all model parameters were adequately estimated and an accurate model was selected in the model comparison, we conducted parameter and model recovery simulations for all RL models, the results of which are provided in the supplementary material.

We used the widely applicable Bayesian information criterion (WBIC; Watanabe, 2013) to assess the model’s goodness of fit. WBIC is the expected value of the inverse log likelihood calculated based on the Markov Chain Monte Carlo (MCMC) samples. All RL models fit the same behavioral data, with the lower the WBIC, the better the data fit. The WBICs calculated from each model were compared to determine which model was the most suitable for the data. The analysis of all computational modeling analyses was done in R (R Core Team, 2021).

We then assessed the Bayes factor to figure out which model had the highest probability of data (BF). Specifically, the BF was determined as the ratio of the marginal likelihood of two models, with the numerator being the marginal likelihood of the model used to generate the data. Kass and Raftery (1995) classified a Bayes factor range of 1–3 as unimportant, 3–20 as positive, 20–150 as strong, and more than 150 as very strong. *BF*_*23*_ = 110, for example, calculated with Model 2’s marginal likelihood as the numerator and Model 3’s marginal likelihood as the denominator, is seen as significant evidence in favor of Model 2 over Model 3.

The raw data and code supporting the conclusions of this paper will be made available without restriction by the authors.

## Results

### Chosen Frequencies in the Preference Judgment Tasks

In the preference judgment tasks, the chosen frequency of high preference stimuli (*M* = 0.68, *SD* = 0.08) was significantly higher than that of low preference stimuli (*M* = 0.09, *SD* = 0.04) (*t*(41) = 37.55, *p* < .001, *d* = 9.58), confirming that there was a difference in preference between the high and low preference stimuli.

### Correct Response Rate in the Gambling Tasks

**Fig. 2:**
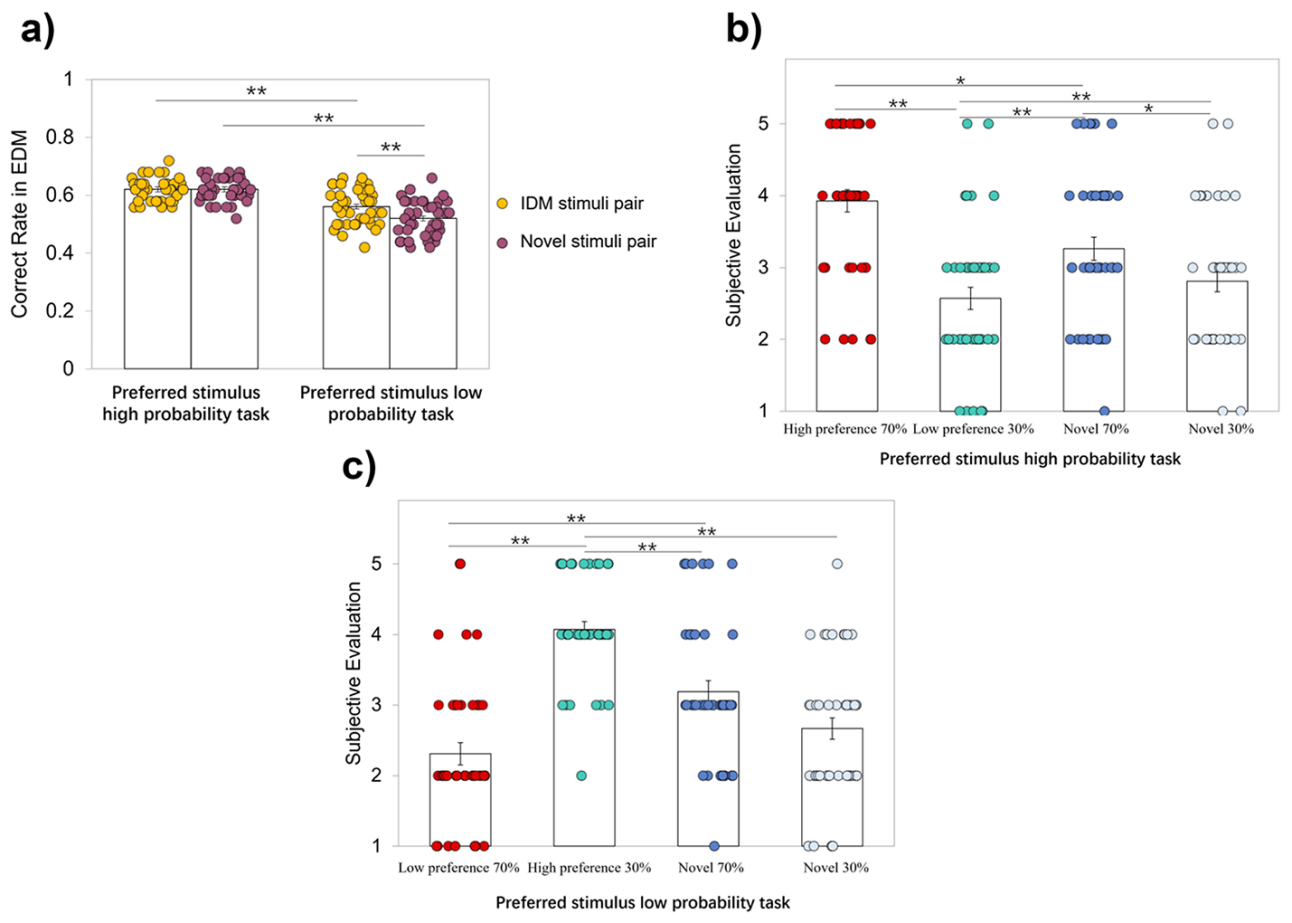
Results of behavioral data. (**a**) The mean correct response rate of all stimuli pairs in the gambling tasks. The preferred stimulus high probability task is one in which the most preferred IDM stimulus is rewarded with a high probability (70%) in EDM. The preferred stimulus low probability task is one in which the most preferred IDM stimulus is rewarded with a low probability (30%) in EDM. (**b**) and (**c**) consist of subjective preference evaluations for four stimuli in each gambling task, encompassing both high and low preference stimuli, as well as novel stimuli. All subjective preferences were rated on a 5-point Likert scale (1 = Extremely Dislike, 5 = Extremely Like). The error bars and colored dots indicate SE and each participant’s data, respectively. * *p* < .05, ** *p* < .001

To investigate whether preferences formed in IDM affect EDM, we compared the correct response rates of all stimulus pairs in the gambling tasks. We conducted a two-factor repeated measures ANOVA for the gambling task type (preferred stimulus high probability task, and preferred stimulus low probability task) and the stimulus type (stimulus presented in the IDM, novel stimulus), and found a significant interaction (*F*(1, 41) = 4.29, *p* < .05, *η*_*p*_^2^ = 0.10). In the preferred stimulus high probability task, the correct response rate of IDM stimuli (0.62) was higher than in the preferred stimulus low probability task (0.56) (Fig. 2a; *F*(1, 82) = 36.23, *p* < .01, *η*_*p*_^2^ = 0.47). Furthermore, for all stimulus pairs, the correct response rate was greater than 0.5 of chance level (*t*s(41) > 2.12, *p*s < 0.05). These results show that when the value of a preferred stimulus is low in EDM, the correct response rate of EDM is lower than that of other situations, which makes it clear that preferences in IDM impact EDM. They also show that when preferences are consistent with the value of learning in EDM (i.e., stimuli with high preferences gain high value in EDM), it is easier to judge which stimulus is the correct answer.

### Subjective Preference Evaluation

We investigated whether stimuli with high preferences formed in IDM are subjectively preferable to novel stimuli after learning in EDM. Multiple comparisons were conducted for the subjective preference of four stimuli in each gambling task. As a result, regardless of the type of gambling task, the preferred stimulus in IDM had the highest subjective evaluation (*t*s(41) > 1.09, *p*s < 0.05, *d*s > 0.24; Fig. 2b, c). These results indicate that the preferences formed in IDM were not affected by learning based on reward feedback in EDM and maintained.

Regarding novel stimuli, the subjective evaluation of high probability reward stimuli was higher than that of low probability reward stimuli in the preferred stimulus high probability task (*t*(41) = 2.95, *p* = .01, *d* = 0.42). In contrast, such results were not found in the preferred stimulus low probability task (*t*(41) = 2.31, *p* = .05, *d* = 0.53). These results indicate that the value of novel stimuli learned in EDM is reflected in subjective preferences only in the easy preferred stimulus high probability task, which includes trials in which the stimulus preferred in IDM receives a high probability of reward in EDM.

### Computational Model Analyses for Actual Experimental Data

A computational model analysis was conducted to examine whether the initial values in the EDM differ between the novel stimuli and the stimuli that have formed preferences in IDM. After fitting the actual EDM behavioral data, which consists of integrated data from the gambling tasks to all models (see Table 2), it was evident from the comparison of the four models that the RL 2 model, in which the initial value of the high preference stimulus in the IDM was different from the other stimuli, showed the best fit to the behavioral data.

**Table 2:** WBIC results of EDM behavioral data fit with four types of RL models

Model	WBIC
RL 1	2189.52*
**RL 2**	**2174.51**
RL 3	2194.00*
RL 4	2185.79**

*Note* * denotes the range of BF values (20 < *BF < 150, 150 < **BF), indicating the degree to which the model that best fits the actual behavioral data is better than the other asterisked models. The bold text represents the model that best fits the behavioral data. The numbers in the table represent the calculated WBIC values

**Fig. 3:**
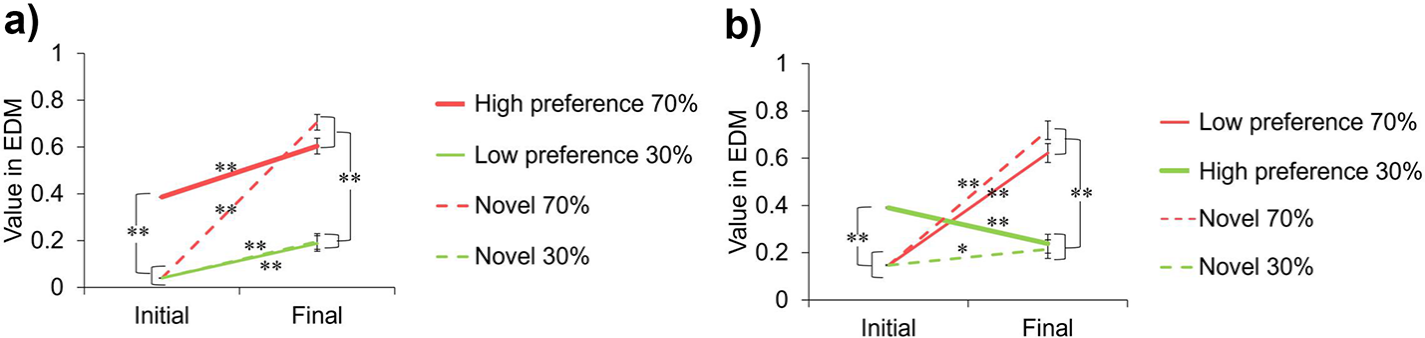
Values of each stimulus estimated by the RL 2 model. (**a**) Preferred stimulus high probability task. (**b**) Preferred stimulus low probability task. The red and green lines are the stimuli with a high (70%) and low (30%) probability of reward in EDM, respectively. The straight and dotted lines are the stimuli that are used in the IDM and novel stimuli in the EDM, respectively. High and low preferences are the most and least preferred stimuli in IDM, respectively. The bold lines are the preferred stimuli in the IDM (i.e., High preference stimuli). Error bars indicate SE. * *p* < .05, ** *p* < .01

Using the RL 2 model, we estimated the value of each stimulus in the gambling tasks and then investigated how the value of IDM is reflected in the initial value of EDM and how it affects subsequent changes in EDM values and even the final value of EDM (see Fig. 3). For each gambling task. We conducted a three-factor repeated measures ANOVA for the stimulus type (stimulus presented in the IDM, novel stimulus), reward probability (high and low), and value types (initial and final). For both gambling tasks, there were significant differences between the initial and final values in all conditions of the combined stimulus type and reward probability (*F*s(1, 41) > 6.99, *p*s < 0.05, *η*_*p*_^2^s > 0.15). Moreover, although the stimuli that had a high value in IDM were more valuable than the other stimuli in terms of the initial values of EDM (*F*s(1, 82) > 467.62, *p*s < 0.01, *η*_*p*_^2^s > 0.92), there was no effect in the final values. In the final values of EDM, only the effect of reward probability remains (*F*s(1, 41) > 166.26, *p*s < 0.01, *η*_*p*_^2^s > 0.80). These results indicate that the higher preference formed in IDM is reflected in the initial values in EDM. Nevertheless, at the end of EDM, the effect of preferences in IDM were not retained after the participant learned values through EDM.

## Discussion

This study aimed to determine whether preferences formed through IDM affect decisions in gambling tasks and whether such preferences are altered after value learning through EDM in gambling tasks.

In gambling tasks, by comparing the correct response rates of stimulus pairs presented in the preference judgment tasks and novel stimulus pairs, we showed that when the preferred stimulus is of lower value in EDM, the correct response rate of gambling tasks decreases due to the inconsistency between the preference and reward in gambling tasks (see Fig. 2a). From this result, preferences formed through IDM impact decision-making behavior (i.e., EDM) in gambling tasks. Additionally, by comparing the fitness of the actual behavioral data to each model, we found that only the RL 2 model with different initial values of the preferred stimulus in IDM is suitable for the behavioral data (see Table 2), and the initial values of the preferred stimulus in the EDM are higher than those of other stimuli (see Fig. 3). Thus, the high preferences formed in IDM are reflected in the initial values of EDM. However, after value learning in EDM, preferences reflected in the initial value of EDM were not retained at the end of the gambling task (see Fig. 3).

These results suggest that while high values in internal criteria guide decision-making behavior in gambling tasks at a stage when the external criteria have not been yet learned. However, the role of internal value in the gambling tasks ends when more external criteria are learned. More specifically, because the preferred stimuli in IDM have higher initial values in EDM, in the preferred stimulus high probability task, where the stimulus with high preference had a high reward probability in EDM, the preferred one was chosen in the early trials without sufficient learning. In fact, on the first trial in which the stimulus pair was presented, 83% of participants chose the high preference 70% stimulus (cf., 66% of participants chose the novel 70% stimulus in the first trial of the novel stimulus pair), which made it easier for participants to learn the stimulus with high reward probability and resulted in a relatively high correct response rate. On the other hand, in the preferred stimulus low probability task, where the preferred stimulus in IDM had a low reward probability in EDM, the choice of a preferred stimulus resulted in choosing a low reward probability in the early gambling task trials. In fact, in the first trial where the stimulus pair was presented, 83% of participants selected the high preference 30% stimulus (cf., 60% of participants chose the novel 30% stimulus in the first trial of novel stimulus pairs), which delayed learning in EDM and made the task relatively difficult. However, as the external criteria become clearer through the learning based on reward feedback in EDM, decisions are made in accordance with the external criterion and are no longer affected by preferences in both gambling tasks.

Interestingly, the stimuli that were most preferred in IDM were also most preferred after the value learning in EDM, regardless of whether they were assigned to high or low probability rewards in the gambling tasks (see Fig. 2b, c), indicating that external and internal criteria differ. Although the results of the computational model analysis showed that the effect of high values (preferences) in internal criteria on external criteria is attenuated by RL in EDM (see Fig. 3b), high subjective preferences were maintained even after learning the values of EDM. If the internal and external criteria were the same, the preference evaluation of stimuli preferred in IDM would decrease after the preferred stimulus low probability task. However, the preference evaluation of those stimuli was still high after the gambling task, that is, the reward feedback in gambling tasks does not alter pre-existing preferences. This suggests that external and internal criteria are distinctively represented in our mind and brain, which is consistent with Ugazio et al.’s (2021) assertion that although monetary value (i.e., value on external criteria) and moral preference (i.e., value on internal criteria) use a similar principle to calculate the value, they are reflected in separate domain-specific brain regions.

Unlike previous studies (Zhu et al., 2021, 2024), the present study showed the preferences learned in IDM formed from the choice behavior were consistent with the subjective preferences. In other words, items frequently chosen in the IDM’s preference judgments were also rated highly in the subjective preference evaluation. Different from previous research, we presented the most and least preferred stimuli in the IDM in a fixed combination in the gambling tasks. Given that this study demonstrated that preferences influence judgments in the early stages in the gambling tasks, it is likely that the participants were considered to have been strongly impressed by their preferences during the EDM learning phase. Thus, our results suggest that if participants retain their impressions of preferences for the stimuli, the preferences formed from their choice behavior show consistency with their subjective preferences. The inconsistencies between preference choices and subjective preferences in previous studies (Zhu et al., 2021, 2024) were likely due to the experimental paradigm they used, namely the presentation of a large number of stimuli and their repeated appearance without the same combination, which likely made it difficult for participants to retain impressions sufficient to reflect subjective preferences.

Additionally, novel stimuli with high probability rewards had a high subjective preference (see Fig. 2b, c). Our results reveal that high values learned through EDM alone are reflected in subjective preferences; that is, reward feedback in gambling tasks influences the formation of preferences for stimuli without clear preferences. In other words, the values learned in EDM impact the internal criteria. This can also be considered part of the internalization process of values learned through external criteria on the premise of no obvious preferences. Combined with the analysis of the computational models in the EDM, there appears to be an interactive relationship between the external and internal criteria, which may also be relevant to a common neural substrate function in both EDM and IDM (Izuma et al., 2010; Miyagi et al., 2017; Nakao et al., 2016).

Overall, we show a novel possibility for a relationship between IDM and EDM: although there is a distinction between the internal and external criteria, they influence each other. However, the present study has several major limitations. First, the preferences in this study were formed through the free choice of novel contour shapes aimed at exploring the most fundamental general relationship between preferences and decision-making in gambling tasks. Therefore, the present findings may not fully reflect the influence of real-life preferences on gambling tasks.

Second, while the novel contour shapes used in this study have been widely employed in EDM and IDM research to mitigate initial value differences between stimuli (Ohira et al., 2009, 2010; Zhu et al., 2021, 2024), it cannot be ruled out that participants may have differed in their subjective attractiveness upon initial exposure to these stimuli.

Third, the results of the model comparison revealed the low preferences in IDM are not reflected in the initial values of EDM. This may be due to the setting of the preference judgment task used in this study, in which a large number of stimuli were presented, and the stimulus combination varied from trial to trial. Therefore, under such a high cognitive load, it is difficult to make an impression of the unselected stimuli in IDM, which cannot be reflected in the initial EDM value. Suppose the preference judgment task is like the gambling task, which is presented multiple times with a fixed and small number of stimulus pairs. In that case, it is conceivable that the unselected stimuli could also leave an impression and be reflected in the initial EDM values.

Fourth, in the gambling task, when participants selected a correct answer, they were rewarded 1 point, which increased their monetary rewards, but when a wrong answer was chosen, the feedback was “0,” which did not decrease their monetary reward. This can be related to the fact that even though the preferred stimulus in IDM did not receive a reward in EDM, it was still able to maintain a high subjective preference after the gambling task. Additionally, gambling in everyday life involves the loss of points or money. Not receiving a reward is different from losing money or points one has. Therefore, gambling tasks involving the loss of money should also be tested.

Fifth, further confirmation as to whether external and internal criteria are expressed differently is desirable. Although the results of the subjective preference evaluation indicated that the EDM and IDM criteria are independent, the subjective and behavioral preferences were inconsistent in previous studies (Zhu et al., 2021, 2024), contradicting the findings of this study. Thus, it is preferable to validate our results with behavioral data, not only subjective preference evaluation. For example, in the preferred stimulus low probability task, the stimulus with the highest value (preferred) in IDM was assigned to the low reward probability stimuli in EDM. If the stimulus maintains its high value in IDM when that stimulus is presented again in IDM, behavioral data and computational model analysis can confirm the uniqueness of the value in IDM.

## Conclusion

In this study, we discovered that high preferences in IDM influence the initial behavior in gambling tasks. Although monetary rewards in the gambling tasks cannot change participants’ strong preferences in IDM, they can form new ones. Therefore, in this study, we show for the first time a new possibility of the relationship between EDM and IDM; that is, the values between EDM and IDM differ but the value learned in IDM can impact EDM. This new relationship is expected to further promote a comprehensive understanding of the decision-making process.

## Electronic Supplementary Material

Below is the link to the electronic supplementary material.

Supplementary Material 1

## Data Availability

The raw data and code supporting the conclusions of this paper will be made available without restriction by the authors.
