# Supplementary material for: Internally Formed Preferences for Options only Influence Initial Decisions in Gambling Tasks, while the Gambling Outcomes do not Alter these Preferences
Source: J Gambl Stud. 2024 Jun 26;41(2):789–803. doi: 10.1007/s10899-024-10326-2 (PMC12116878; doi:10.1007/s10899-024-10326-2)
Supplement: Supplementary file 1 — Supplementary Material 1 [file 10899_2024_10326_MOESM1_ESM.pdf]

Internally formed preferences for options only influence initial decisions in gambling tasks, while the gambling outcomes do not alter these preferences

Supplemental Materials

To verify whether the model used in this study conforms to the experimental design of this study, all RL models were simulated for parameter and model recovery (Wilson & Collins, 2019).

Parameter recovery

In parameter recovery, we investigated whether model parameters used to generate artificial behavioral data could be estimated by fitting the model to the artificial data. Pearson's correlation coefficient was determined between the simulated and estimated parameters to calculate parameter recovery indices. In this study, models were considered capable of complete parameter recovery if the correlation coefficients were greater than 0.5. The artificial behavioral dataset was generated using the same settings as the actual experimental design of the gambling tasks. We created artificial data for each model using four stimuli and 100 trials for 42 participants.

The estimation of model parameters is completed in R (R Core Team, 2021) using the hierarchical Bayesian method. The parameters for each participant were generated by a shared distribution within the group. As shown in Equation s1, the normal distributions of μ and σ^2 were thought to have created parameter θ (e.g., α, β) of one participant. For the prior distributions of μ and σ^2, we used the uniform distribution. Additionally, we trimmed the normal distribution to ensure that the generated parameters fell within a specific range. The population-level distribution parameters took a prior distribution into consideration, which generated a posterior

distribution using the Bayes estimator. The posterior distribution of parameters was calculated using the MCMC method.

Equation s1 $\theta \sim N(\mu_\theta, \sigma_\theta^2)$

In every model, the normal distribution of μ and σ^2 was used to generate the parameters of α ($0 \leqq \alpha \leqq 1$), β ($0 \leqq \beta \leqq 20$), and η ($0 \leqq \eta \leqq 1$). μ_β and σ_β^2 were generated from the uniform distributions of [0, 20] and [0, 10], respectively, while μ_α, σ_α^2, μ_η and σ_η^2 were all generated from the uniform distribution of [0, 1].

By applying the artificial data to the computational model used to generate it, we confirmed that the parameters of the computational model set (simulated) at the time of the artificial data generation could be appropriately estimated. The results, shown in Figure s1, indicate that the simulated and estimated values from all models were consistent to some extent ($r > .52$).

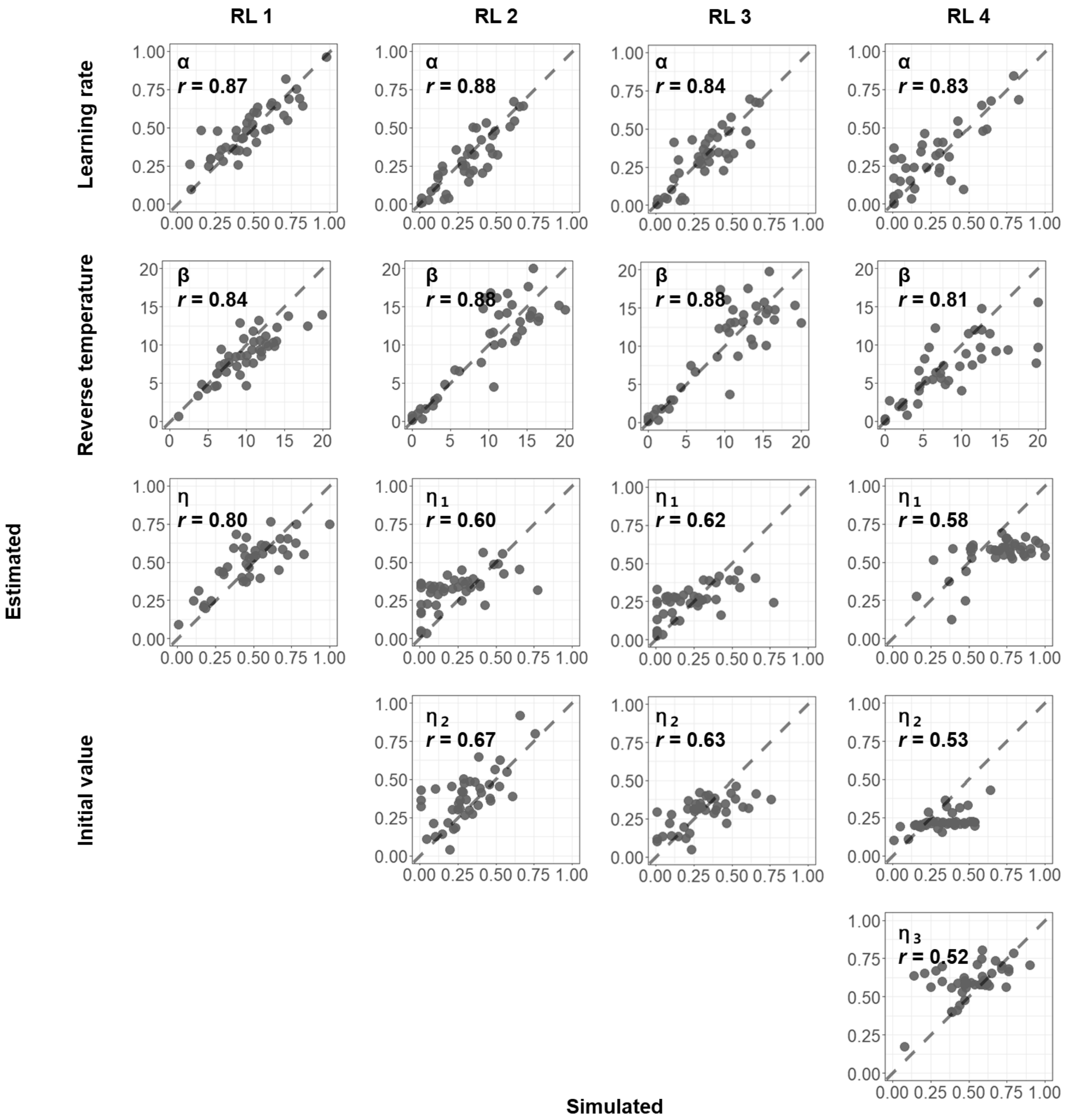

Figure s1. Simulation results

The purpose of this simulation was to determine whether each model could estimate the values of each parameter. As indices, the Pearson's correlation coefficient between simulated and fitted data was displayed.

Model recovery

Model recovery is performed to test whether the true model is the most fit to the data generated by that model under the experimental design. We applied the dataset generated from each model to all models and evaluated the relative goodness of fit of the models using the WBIC (Watanabe, 2013). The BF values between each model were also calculated to compare the differences between the models.

The model recovery results are shown in Table s1. When RL 2, 3, and 4 were true models (models used to generate artificial data), the best fit was obtained when the analysis was performed with the same models as the true models, confirming that model recovery is possible for these models. However, when RL 1 was a true model, RL 3 was more appropriate than other models. Thus, if RL 3 is used as the model that best fits the behavioral data, it is impossible to distinguish which of the RL1 and RL 3 models is the true one based on the data obtained from the experimental settings in this study. However, note that RL 2, the model that best fits the behavioral data in this study, can complete model recovery.

Table s1

Result of model recovery

Simulated \ Estimated	RL 1	RL 2	RL 3	RL 4
RL 1	2170.12**	2153.48**	**2147.94**	2150.84^{+}
RL 2	1998.94**	**1991.47**	2004.64**	1999.87**
RL 3	2142.79**	2123.62**	**2098.62**	2109.50**
RL 4	2152.68**	2151.49**	2144.55^{+}	**2143.42**

Note: "Simulated" indicates the model for which artificial data was generated, and "Estimated" indicates the model used in the computational model analysis. */$^{+}$ is the range of BF values (3 < $^{+}$*BF* < 20, 150 < ***BF*), indicating that the model with the best fit was favored over

the other models when the model for which the virtual data was generated was the true model. Bolded text indicates the model that best fits the hypothetical data. The numbers in the table indicate the calculated WBIC values.

References

R Core Team. (2021). R: A language and environment for statistical computing. https://www.r-project.org/. Vienna, Austria: R Foundation for Statistical Computing.

Wilson, R. C., & Collins, A. G. (2019). Ten simple rules for the computational modeling of behavioral data. *elife*, *8*, e49547. https://doi.org/10.7554/eLife.49547
